# Natural Compounds That Activate the KEAP1/Nrf2 Signaling Pathway as Potential New Drugs in the Treatment of Idiopathic Parkinson’s Disease

**DOI:** 10.3390/antiox13091125

**Published:** 2024-09-18

**Authors:** Sandro Huenchuguala, Juan Segura-Aguilar

**Affiliations:** 1 Escuela de Tecnología Médica, Facultad de Salud, Universidad Santo Tomás, Santiago 8370003, Chile; shuenchuguala@santotomas.cl; 2 Molecular & Clinical Pharmacology, Instituto de Ciencias Biomédicas (ICBM), Faculty of Medicine, University of Chile, Santiago 8380453, Chile

**Keywords:** Parkinson’s disease, KEAP1/Nrf2, dopamine, neurodegeneration, neuroprotection, single-neuron degeneration, aminochrome, preclinical model, dopaminergic neurons, neuromelanin

## Abstract

Recently, a single-neuron degeneration model has been proposed to understand the development of idiopathic Parkinson’s disease based on (i) the extremely slow development of the degenerative process before the onset of motor symptoms and during the progression of the disease and (ii) the fact that it is triggered by an endogenous neurotoxin that does not have an expansive character, limiting its neurotoxic effect to single neuromelanin-containing dopaminergic neurons. It has been proposed that aminochrome is the endogenous neurotoxin that triggers the neurodegenerative process in idiopathic Parkinson’s disease by triggering mitochondrial dysfunction, oxidative stress, neuroinflammation, dysfunction of both lysosomal and proteasomal protein degradation, endoplasmic reticulum stress and formation of neurotoxic alpha-synuclein oligomers. Aminochrome is an endogenous neurotoxin that is rapidly reduced by flavoenzymes and/or forms adducts with proteins, which implies that it is impossible for it to have a propagative neurotoxic effect on neighboring neurons. Interestingly, the enzymes DT-diaphorase and glutathione transferase M2-2 prevent the neurotoxic effects of aminochrome. Natural compounds present in fruits, vegetables and other plant products have been shown to activate the KEAP1/Nrf2 signaling pathway by increasing the expression of antioxidant enzymes including DT-diaphorase and glutathione transferase. This review analyzes the possibility of searching for natural compounds that increase the expression of DT-diaphorase and glutathione transferase through activation of the KEAP1/Nrf2 signaling pathway.

## 1. Parkinson’s Disease

Parkinson’s disease is the second most prevalent neurodegenerative disease, and its main symptoms are tremors, muscle rigidity and bradykinesia. Seventy percent of people with Parkinsonism have idiopathic Parkinson’s, which is characterized by an onset after 55–60 years of age [1]. The identity of the neurotoxin that triggers the degenerative process of dopaminergic neurons containing neuromelanin is unknown. Although the identity of the neurotoxin that triggers the degenerative process in idiopathic Parkinson’s is unknown, there is a consensus within the scientific community that mitochondrial dysfunction, alpha-synuclein aggregation, dysfunction of both lysosomal and proteasomal protein degradation systems, endoplasmic reticulum stress, neuroinflammation and oxidative stress are involved in the neurodegenerative process [2,3,4,5,6,7,8,9]. In 20% of all Parkinson’s patients, the cause that triggers the degenerative process is known. Parkinsonism inducers in this group include metals such as manganese and copper; pesticides such as paraquat; certain drugs (such as antidepressants, calcium channel antagonists, cholinomimetics, antiemetics, anti-vertigo drugs, antiarrhythmics and antiepileptic drugs) and traumas such as head injuries received during boxing [10,11]. Ten percent of Parkinson’s patients have genetic Parkinson’s in which the cause that triggers the degenerative process is a mutation in a gene such as alpha-synuclein, parkin or LRRK2, among other genes [12].

## 2. Clinical Studies of Parkinson’s Disease

The discovery of the decrease in dopamine levels resulting from the loss of neuromelanin-containing dopaminergic neurons in the substantia nigra has been one of the most important discoveries in research on idiopathic Parkinson’s disease [13]. This drop in dopamine levels is related to the appearance of motor symptoms when 60% of the neuromelanin-containing dopaminergic neurons in the nigrostriatal system have been lost. Given this drop in dopamine levels, the idea arose that by replacing the patient’s dopamine levels, the motor symptoms could disappear. In the 1960s, the first clinical studies began with the dopamine precursor L-dopa, which, unfortunately, were not successful because the dose of L-dopa was not high enough. However, as early as 1967, L-dopa had been incorporated into the treatment of the disease. The results of L-dopa are excellent at the beginning because the patient recovers a large proportion of normal mobility, which allows the individual to live a relatively normal life. However, after chronic treatment for 4–6 years, severe secondary symptoms appear, such as dyskinesias, which have a devastating effect on the patient’s normal life [14].

It took a few years to develop a drug for the pharmacological treatment of Parkinson’s disease once it was clear what the treatment needed to accomplish (restore dopamine levels). In 57 years of intense basic research, several molecules were identified that could have a potential therapeutic effect in idiopathic Parkinson’s disease. The potential therapeutic effect of these drugs was based on successful preclinical studies using exogenous neurotoxins such as 6-hydroxydopamine and MPTP (1-methyl-4-phenyl-1,2,3,6-tetrahydropyridine) [15,16,17,18]. These successful preclinical studies included drugs such as isradipine, mitoquinone, coenzyme Q10, zonisamide, TCH346, nilotinib, deferiprone, prasinezumab, cinpanemab and neurturin [19,20,21,22]. The most promising drug is neurturin, an analogue of glial cell-derived neurotrophic factor (GDNF), which showed spectacular effects and could even be used in patients with L-dopa dyskinesias since they regenerated neuronal tissues in preclinical studies with 6-hydroxydopamine [23,24]. However, the translation of these successful preclinical studies to clinical studies and new drugs for the treatment of idiopathic Parkinson’s disease has failed. The question is why all these clinical studies failed. The authors of the phase 3 clinical trial of mitoquinone explained that the failure of the study depended on several factors: (i) The study had methodological problems such as an inadequate number of patients. (ii) By the time symptoms appear, more than 50% of the dopaminergic neurons containing neuromelanin have been lost. Therefore, the fate of the surviving neurons is already decided, and they are eventually lost regardless of treatment with mitoquinone. (iii) The penetration of mitoquinone into the brain is insufficient to have a neuroprotective effect. (iv) Oxidative damage to mitochondria is not the main reason for the loss of dopaminergic neurons containing neuromelanin, and therefore, treatment with mitoquinone cannot stop the progression of the disease [21]. The authors of the phase 3 clinical trial of coenzyme Q10 suggested that the failure of the study was due to the fact that the diagnosis of the disease is made when more than 50% of the neuromelanin-containing dopaminergic neurons have been lost and that it is already too late to start treatment with coenzyme Q10; treatment should be started before the onset of motor symptoms [20]. A review has recently been published that thoroughly analyzes the antioxidant therapy of Parkinson’s disease and suggests that the failure of clinical studies depends on two factors: (i) the animals used in preclinical studies with antioxidants were not aged animals; (ii) the diagnosis of patients was based on clinical criteria in which symptoms appear when 60–80% of the dopaminergic neurons containing neuromelanin have been lost, due to the lack of molecular diagnosis [25]. In our opinion, this depends on preclinical models based on exogenous neurotoxins such as 6-hydroxydopamine and MPTP that do not represent the neurodegenerative process that occurs in the nigrostriatal system in idiopathic Parkinson’s disease. These preclinical models with exogenous neurotoxins such as MPTP and 6-hydroxydopamine induce extremely rapid Parkinsonism in animals. In humans, we only know the effect of MPTP, which induces severe Parkinsonism in just 3 days [26], which contrasts with the extremely slow progress of idiopathic Parkinson’s disease.

It has been proposed that the degenerative process of the nigrostriatal system may take years before motor symptoms appear, which occurs when 60% of the dopaminergic neurons containing neuromelanin have been lost. After the onset of motor symptoms, progress can take 10–15 years. For example, Pope John Paul II died after suffering for 13 years from idiopathic Parkinson’s; he was diagnosed in 1992, when he was 71 years old, and died in 2005. A study has recently been published that estimates the number of neuromelanin-containing dopaminergic neurons in the substantia nigra. Considering the two hemispheres of the human brain, it was determined that there are 1,000,000–800,000 dopaminergic neurons that contain neuromelanin [27]. This implies that at the time of the appearance of motor symptoms when 60% of these neurons have disappeared, there should be 400,000–320,000 surviving neurons. This implies that in a patient who survives 15 years after the onset of motor symptoms, 73–58 neuromelanin-containing dopaminergic neurons are lost per day. In the clinical study of mitoquinone, a powerful mitochondrial antioxidant, two oral doses were administered over 12 months [21]. In the best of cases, mitoquinone has a 100% effect, stopping the loss of 26,645–21,170 dopaminergic neurons containing neuromelanin. In the case that mitoquinone has a 50% neuroprotective effect, 13,322–10,585 dopaminergic neurons containing neuromelanin will survive after this treatment. The question is whether the Unified Parkinson Disease Rating Scale is sensitive enough to detect a slowing of disease progression under these conditions. The extremely slow progress of the disease could explain the failure of these clinical studies because the therapeutic effect of the drug is limited to a very small number of neurons that degenerate every day. In contrast, the positive effect observed in preclinical studies was achieved with exogenous neurotoxins that induce a rapid and massive effect and are also not responsible for the degenerative process in the brain of the patient with idiopathic Parkinson’s.

## 3. The Role of Endogenous Neurotoxins in Idiopathic Parkinson’s Disease

The extremely slow progress of the degenerative process of idiopathic Parkinson’s disease suggests that (i) the degenerative process before and after the appearance of motor symptoms cannot be induced by exogenous neurotoxins due to its massive and extremely rapid character, and (ii) the neurotoxin that triggers the degenerative process in idiopathic Parkinson’s disease must be generated within dopaminergic neurons that contain neuromelanin and cannot have an expansive character that affects neighboring neurons. The degenerative process seems to be individual, affecting only the neuron that generates this endogenous neurotoxin, explaining the extremely slow loss of dopaminergic neurons that contain neuromelanin. Recently, a single-neuron degeneration model has been proposed for idiopathic Parkinson’s disease based on these ideas [28].

Among the endogenous neurotoxins that are formed within dopaminergic neurons that contain neuromelanin, alpha-synuclein is one of the best candidates. It has been proposed that alpha-synuclein may exert its neurotoxic effect by aggregating to form fibrils that are deposited in Lewy bodies or oligomers. Alpha-synuclein induces oxidative stress, mitochondrial dysfunction, neuroinflammation, lysosomal dysfunction, autophagy impairment, synaptic dysfunction and proteasome impairment [2,3,4,5,6,7,8,9]. However, both the fibrils and their oligomers have been reported to have an expansive character [9,29,30,31,32,33]. The concept of a propagative neurotoxin refers to its ability not only to induce neurotoxicity and death in the neuron where it acts but also to affect neighboring neurons through the secretion of this neurotoxin. Alpha-synuclein can exert a neurotoxic effect by forming neurotoxic oligomers and inducing death of a specific neuron. It has been shown that oligomers of alpha-synuclein can be secreted, affecting neighboring neurons, generating a propagative effect in which a single neurotoxic oligomer affects many neurons. Alpha-synuclein can also form fibrils that accumulate in Lewy bodies, which have been reported to have a propagative effect, since they propagate from one brain region to another [9,29,30,31,32,33]. This expansive character of alpha-synuclein aggregation implies that it is not suitable for the single-neuron degeneration model.

Another endogenous neurotoxin formed within dopaminergic neurons is 3,4-dihydroxyphenylacetaldehyde (DOPAL), generated in the oxidative deamination of dopamine mediated by monoamine oxidase [34]. In a study carried out with postmortem material from patients with Parkinson’s disease, a low expression of the enzyme aldehyde dehydrogenase-1 was observed, suggesting that a low expression of this enzyme would end in an accumulation of DOPAL [35]. However, the low expression of aldehyde dehydrogenase-1 was observed in postmortem material that survived the degenerative process for years with Parkinson’s disease. DOPAL induces the formation of alpha-synuclein oligomers, apoptosis and oxidative stress and affects mitochondrial function [36,37]. DOPAL also has an expansive character based on its transmissibility from dopaminergic neurons to glial cells, which makes it incompatible with the single-neuron degeneration model [38].

Aminochrome is a transient endogenous neurotoxin that is formed during the synthesis of neuromelanin. Neuromelanin is formed from the oxidation of the catechol group of dopamine to ortho-quinone dopamine, which, at physiological pH, cyclizes to amino-like immediately at a rate of 0.15 s^−1^. Aminochrome is also not stable, but in in vitro NMR experiments it is stable for 40 min before converting to 5,6-indolequinone at a rate of 0.06 min^−1^, which rapidly polymerizes, forming neuromelanin [39]. The characteristic of being a transient metabolite in the synthesis of neuromelanin suggests that aminochrome cannot generate expansive neurotoxicity to neighboring neurons. It is impossible that aminochrome in the cytosol of a neuromelanin-containing dopaminergic neuron full of proteins, lipids and biomolecules can be stable for 40 min. Aminochrome can be rapidly reduced by flavoenzymes that transfer one electron to the aminochrome *o*-semiquinone radical, which is extremely reactive with oxygen [40] or can be reduced by NAD(P)H:quinone oxidoreductase (DT-diaphorase; NQO1; EC 1.6.99.2) with two electrons to leukoaminochrome [41,42]. Alternatively, aminochrome can form adducts with proteins such as actin, alpha- and beta-tubulin, alpha-synuclein and mitochondrial complex I, among other proteins [43,44]. This implies that the formation of aminochrome and its neurotoxic action within the neuromelanin-containing dopaminergic neuron only affects a single neuron. Another interesting characteristic of aminochrome is that its neurotoxicity depends on its ability to induce mitochondrial dysfunction, oxidative stress, aggregation of alpha-synuclein to neurotoxic oligomers, dysfunction of protein degradation of both the lysosomal and proteasomal systems, endoplasmic reticulum stress and neuroinflammation [44,45,46,47,48,49,50,51,52].

Among the neurotoxins that are currently known, aminochrome is the endogenous neurotoxin that is most suitable for the single-neuron degeneration model proposed as a model of neurodegeneration in idiopathic Parkinson’s disease, since its neurotoxic action affects a single neuron. The neurotoxic action of aminochrome affecting a single neuron is consistent with the extremely slow progression of the degenerative process of the nigrostriatal system in idiopathic Parkinson’s disease.

## 4. Aminochrome as a Preclinical Model of Idiopathic Parkinson’s Disease

The use of aminochrome in preclinical models for idiopathic Parkinson’s disease in animals is technically impossible. With current technology, an intracerebral injection of aminochrome in the striatum, substantia nigra or medial forebrain bundle results in the neurotoxic action of aminochrome in all areas where the aminochrome injection reaches, affecting the neurons that have a dopamine transporter [47].

If we consider that aminochrome may be the endogenous neurotoxin capable of inducing the degeneration of a single neuron in idiopathic Parkinson’s disease, the question is how to prevent the neurotoxic action of aminochrome. DT-diaphorase prevents the formation of leukoaminochrome *o*-semiquinone radical when aminochrome is reduced with one electron by flavoenzymes that transfer an electron using NADH or NADPH as the electron donor. The leukoaminochrome *o*-semiquinone radical is extremely reactive with oxygen, generating oxidative stress [40,48]. DT-diaphorase also prevents the formation of aminochrome adducts with several proteins. DT-diaphorase prevents the formation of neurotoxic alpha-synuclein oligomers [44] that have been postulated to play an important role in the degenerative process of idiopathic Parkinson’s disease [53]. DT-diaphorase prevents disruption of the cytoskeleton when aminochrome forms adducts with alpha- or beta-tubulin [43]. Interestingly, astrocytes secrete glutathione transferase M2-2 through exosomes that penetrate dopaminergic neurons, releasing this enzyme into their cytosol [54,55,56]. DT-diaphorase and glutathione transferase M2-2 constitute a neuroprotective mechanism in which astrocytes play an important role by secreting exosomes loaded with glutathione transferase M2-2, which increases the neuroprotection that DT-diaphorase can provide to neuromelanin-containing dopaminergic neurons. This neuroprotective mechanism may explain why neuromelanin synthesis is not neurotoxic in older adults who, at the time of death, have their neuromelanin-containing dopaminergic neurons intact in the substantia nigra even though aminochrome is formed during neuromelanin synthesis [57,58].

## 5. KEAP1/Nrf2 Signaling Pathway

The transcription factor nuclear factor E2-related factor 2 (Nrf2) regulates the expression of a wide range of genes that play a cytoprotective role in the presence of oxidative stress or xenobiotic [59,60]. Normally, the human body is exposed to a series of xenobiotics of external origin through food, drugs, environmental contaminants or illicit drugs. All water-soluble xenobiotics are eliminated through urine, but those that are not water-soluble must be metabolized by Phase 1 enzymes. The goal of Phase 1 enzymes such as cytochrome P450 is to activate these compounds so that Phase 2 enzymes can conjugate them to increase their water solubility, facilitating their elimination [61]. The category of enzymes that conjugate xenobiotics includes glutathione transferases, UDP-glucuronosyltransferases, N-acetyltransferases, sulfotransferases and methyltransferases. During oxidative stress, Nrf2 increases the expression of antioxidant enzymes such as superoxide dismutase, glutathione peroxidase, catalase, heme oxygenase-1 and DT-diaphorase, among others [62].

**Figure 1:**
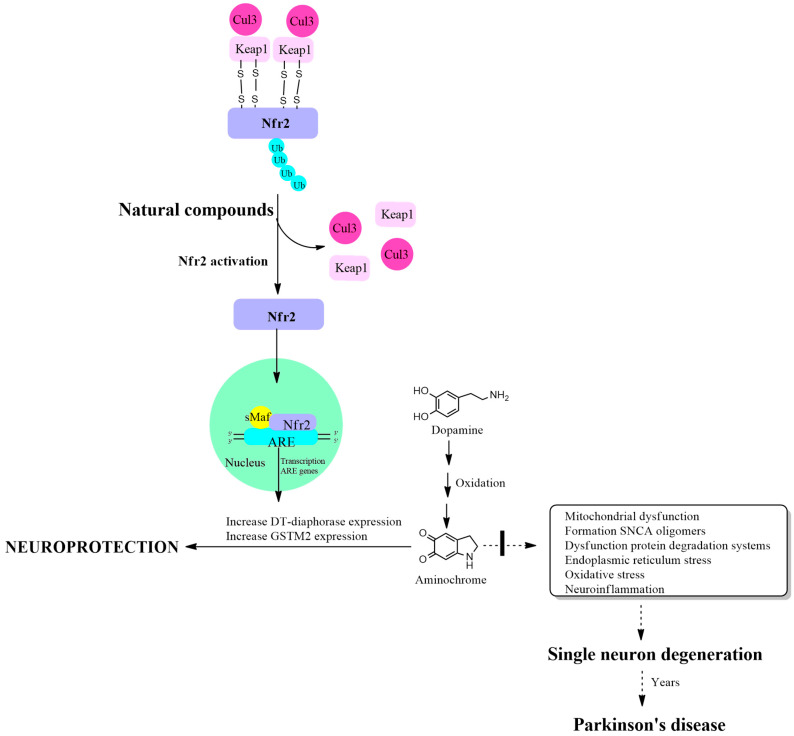
Increased expression of DT-diaphorase, glutathione transferase M2-2 and other antioxidant enzymes through activation of the KEAP1/Nrf2 signaling pathway. Cullin 3 E3 ubiquitin ligase (Cul3); Kelch-like ECH-associated protein 1 (KEAP1); E2-related factor 2 (Nfr2); small musculoaponeurotic fibrosarcoma (sMaf); antioxidant responsive element (ARE).

Nrf2 activation is regulated by the Kelch-like ECH-associated protein 1 (KEAP1) protein, which has a binding site in the Neh2 domain located near the NH3 terminus of the Nrf2 protein. The function of binding two KEAP1 molecules to Nrf2 is to allow their ubiquitination, mediated by Cul3 E3 ubiquitin ligase, for Nrf2 degradation through the proteasomal system. This mechanism maintains very low basal levels of Nrf2 and prevents transcription of antioxidant genes. The KEAP1 protein has two cysteine amino acids located at position 273 and 288 that are oxidized in the presence of oxidative stress, leaving free the Nrf2 protein that activates the transcription of antioxidant genes [63,64]. Another study on the structure of the KEAP1 protein and its cysteine residues revealed that cysteine 151 is also important in the maintenance of the KEAP1/Nrf2 complex, necessary for its ubiquitination, which is required for degradation in the proteasomal system [65]. The cysteine residues in Nrf2 at positions 119, 235 and 506 also play an essential role in the binding of Nrf2 with the antioxidant responsive element for the activation of transcription of antioxidant genes. The mutation of these cysteines prevents the transcription of these antioxidant genes [66] (Figure 1).

## 6. Activation of the KEAP2/Nrf2 Signaling Pathway by Natural Products Increases the Expression of Antioxidant Enzymes

A long list of natural products has been shown to have antioxidant properties mediated by activation of the KEAP1/Nrf2 signaling pathway. Natural products that activate the KEAP1/Nrf2 signaling pathway are present in the following: (i) dietary phytochemicals, including cruciferous vegetables such as broccoli, cauliflower and cabbage that contain sulforaphane, which is marketed as an antioxidant and anti-inflammatory; (ii) fruits such as red grapes that contain resveratrol which is marketed as an antioxidant; (iii) plants such as the species Curcuma longa, the source of curcumin, which is marketed as food flavoring and food coloring; and (iv) carotenoids that contain astaxanthin which is marketed as an antioxidant, anti-inflammatory and UV protectant. The natural compound hyperoside has an antioxidant effect against oxidative stress by activating the KEAP1/Nrf2 signaling pathway, promoting an increase in antioxidant enzymes in testicular tissue [67,68]. Sulforaphane, a natural compound detected in broccoli sprout extracts, has been shown to exert antioxidant and cytoprotective effects by activating Nrf2, which regulates the levels of thyroglobulin, a precursor of thyroid hormones [69,70,71,72]. Natural compounds that we consume in a diet composed of vegetarian products and fruits regulate oxidative stress through the KEAP1/Nrf2 signaling pathway and are important in the prevention and treatment of cancer; examples include resveratrol, curcumin, sulforaphane, quercetin, phenethyl isothiocyanate, epigallocatechin gallate, hesperidin and 2′-hydroxyflavanone [72,73,74,75,76,77,78]. An experiment in PC 12 cells exposed to Aβ25-35-dependent oxidative stress demonstrated the neuroprotective and antioxidant role of two curcumin analogues that increase the expression of superoxide dismutase, catalase and heme oxidase-1 through the activation of the KEAP1/Nrf2 signaling pathway [74]. The natural compound withaferin A, found in the plant *Withania somnifera* activates Nrf2, increases the expression of antioxidant enzymes in cultures of human umbilical vein endothelial cells and in endothelial cell lines [79]. Thonningianin A, a natural compound found in *Penthorum chinense* Pursh, has shown a protective effect against ferroptosis in experiments with SH-SY5Y cells treated with 6-hydroxydopamine by activating the KEAP1/Nrf2 signaling pathway [80]. Berberine, an alkaloid of the isoquinoline type extracted from plants, demonstrated a protective effect against methotrexate-dependent nephrotoxicity accompanied by recovery of reduced glutathione levels and superoxide dismutase activity through the activation of the KEAP1/Nrf2 signaling pathway [81]. A byproduct grape seed meal was shown to counteract the oxidative stress induced by E. coli lipopolysaccharide in IPEC-1 cells and restore the levels of antioxidant enzymes such as catalase, superoxide dismutase and glutathione peroxidase by activating the KEAP1/Nrf2 signaling pathway [82]. The natural product hederagenin, a pentacyclic triterpenoid saponin, extracted from various plant herbs has neuroprotective, anti-inflammatory, anti-cancer, anti-lipid peroxidation and oxidative-stress-mitigating properties through the activation of the KEAP1/Nrf2 pathway [83]. The compound isoglycyrrhizinate extracted from licorice has antioxidant, anti-apoptotic and anti-inflammatory effects. In experiments with animals treated with the hepatotoxicity inducer arsenic trioxide, isoglycyrrhizinate was shown to attenuate oxidative stress through the activation of the KEAP1/Nrf2 signaling pathway [84]. In experiments with the natural probiotic *Clostridium butyricum* that decreases the oxidative effect induced by enterotoxigenic Escherichia coli K88, it was demonstrated that it increases the expression of superoxide dismutase, glutathione peroxidase and decreases oxidative stress through the activation of the KEAP1/Nrf2 signaling pathway [85].

In a study with alloxan induced type 1 diabetes mellitus in mouse-derived pancreatic islet β-cell line, the isoflavonoid formononetin, a component of Astragalus gallinaceus Bunge, activates the KEAP1/Nrf2 signaling pathway, decreasing oxidative stress [86,87]. Physalin H, a compound isolated from the plant *Physalisangulata* L., exerts anti-inflammatory effects by activating the KEAP1/Nrf2 signaling pathway [88]. In a study with diabetes mellitus rats and with the MPC-5 cell line Icarin, a flavonoid extract isolated from *Herba epimedii*, increases mitophagy to inhibit NLRP3 inflammasome activation through the activation of the KEAP1/Nrf2 signaling pathway [89]. A study to determine the mechanism of the pharmacological effect of the alkaloid Oxymatrine, a component of the herb Radix *Sophorae flavescentis*, in the treatment of cardiovascular dysfunctions demonstrated that this effect depends on the activation of the KEAP1/Nrf2 signaling pathway [90]. The extract of the inflorescent *Coptis chinensis* plant, which is used as tea, exerts hepatoprotective effects by activating the KEAP1/Nrf2 signaling pathway, increasing the expression of antioxidant enzymes and reducing oxidative stress induced by carbon tetrachloride [91]. The natural compound withaferin A present in the plant *Withania somnifera* was shown to activate the KEAP1/Nrf2 signaling pathway by increasing the expression of heme oxygenase-1 in an endothelial cell line and primary cultures of human umbilical vein [79]. The antirheumatic drug auranofin increases heme oxygenase-1 expression through activation of the KEAP2/Nrf2 signaling pathway [92]. The activation of transcription of antioxidant enzyme genes mediated by Nrf2 plays a fundamental role in protection against oxidative stress. However, the activation of transcription of antioxidant enzyme genes by Nrf2 can have a negative effect, as in patients with cancer that use drugs where their therapeutic action requires the formation of free radicals that induce oxidative stress [93,94,95].

Experiments in cell cultures with extracts of the herb *Callicarpa kwangtungensis* showed an anti-inflammatory effect mediated by the activation of the KEAP1/Nrf2 signaling pathway, increasing heme oxygenase-1 and DT-diaphorase expression [95]. Oxyresveratrol, a natural compound present in mulberry, which has anti-inflammatory and antioxidant effects, in experiments on mice with liver injury demonstrated a hepatoprotective effect by reducing the activity of aspartate transaminase, alanine transaminase, oxidative stress, expression of inflammatory factors and increasing the expression of heme oxygenase-1 and DT-diaphorase through the activation of the KEAP1/Nrf2 signaling pathway [96]. In experiments with drug-induced liver injury in mice monoammonium glycyrrhizinate, a component of the plant Glycyrrhiza, in combination with cysteine hydrochloride protect against drug-induced liver injury in mice through the activation of the KEAP1/Nrf2 signaling pathway that decreases the oxidative stress and increase expression of heme oxygenase-1 and DT-diaphorase [97]. Shufeng Jiedu has been used in traditional Chinese medicine for decades to treat respiratory conditions. An anti-inflammatory effect accompanied with an increase in the expression of glutathione transferases, heme oxygenase-1, superoxide dismutase, DT-diaphorase through the activation of the KEAP1/Nrf2 signaling pathway was observed in experiments with rats treated with Shufeng Jiedu [98]. Ginnalin A, a natural compound from red maple, activates the KEAP1/Nrf2 signaling pathway, increasing the expression of the enzymes DT-diaphorase, heme oxygenase and the glutamate-cysteine ligase catalytic subunit. Pretreatment of SH-SY5Y cells with ginnalin A prevents 6-hydroxydopamine neurotoxic effects [99]. In experiments with mice presenting acute lung injury, S-allylmercaptocysteine, a compound derived from garlic, demonstrated a protective effect accompanied by a decrease in the production of proinflammatory factors, oxidative stress and an increase in the expression of heme oxygenase-1 and DT-diaphorase through the activation of the KEAP1/Nrf2 signaling pathway [100]. Compound 13f, which contains selenium, was synthesized from verubecestat and ebselen. It has been shown to have a cytoprotective effect against 6-hydroxydopamine and hydrogen peroxide by reducing oxidative stress, apoptosis, mitochondrial damage and calcium overload. An increase in antioxidant enzymes such as heme oxygenase-1 and DT-diaphorase among others was observed through the activation of the KEAP1/Nrf2 signaling pathway [101].

The natural compound carnosic acid, found in the herbs sage and rosemary, increases the expression of antioxidant enzymes by activating the KEAP1/Nrf2 signaling pathway through their conversion to ortho-quinone species during oxidative stress. Synthesized para-hydroquinones increase the expression of antioxidant enzymes such as heme oxygenase-1 and DT-diaphorase through the KEAP1/Nrf2 signaling pathway [102].

Crocin, a natural carotenoid present in the natural compound flower of crocus and gardenia species, activates the KEAP1/Nrf2 signaling pathway by increasing and decreasing oxidative stress by increasing the expression of antioxidant enzymes such as glutathione transferase, superoxide dismutase, catalase and glutathione peroxidase as well as glutathione levels [103]. In brain injury after blood infusion, the KEAP1/Nrf2 signaling pathway is activated, increasing the expression of glutathione transferase, heme oxygenase-1 and the levels of reduced glutathione and thioredoxin [104]. The prenylated flavonoid chalcone xanthohumol, present in spent hops, increases the expression of detoxifying enzymes such as DT-diaphorase and glutathione transferase [105]. *Atractylodis rhizoma*, a Chinese medicinal drug with anti-inflammatory and antiviral properties, activates the KEAP1/Nrf2 signaling pathway, which increases the expression of DT-diaphorase [106]. After subarachnoid hemorrhage during early brain injury tert-butylhydroquinone decreased cognitive dysfunction and damage by activating the KEAP1/Nrf2 signaling pathway that increased the expression of glutathione transferase, DT-diaphorase and heme oxygenase-1 [107]. Safranal, which has anti-apoptotic and antioxidant activity, protects from the neurotoxic effects of rotenone in in vitro experiments by activating the KEAP1/Nrf2 signaling pathway by increasing the expression of glutathione S transferase, DT-diaphorase and heme oxygenase-2. [108]. Dietary antioxidants such as astaxanthin, a carotenoid, and docosahexaenoic acid and eicosapentaenoic acid, found in fish, increase the expression of DT-diaphorase and glutathione transferase M2-2 through the KEAP1/NRF2 signaling pathway [109]. The natural compound Geniposide, isolated from *Gardenia jasminoides* Ellis, induces the expression of glutathione transferase M1-1 and M2-2 probably through the activation of the KEAP1/Nrf2 signaling pathway [110]. The phytochemical berberrubine and resveratrol activate the expression of glutathione transferase M2-2 probably through the KEAP1/Nrf2 signaling pathway(Table 1) [111,112].

## 7. Activation of the KEAP2/Nrf2 Signaling Pathway by Natural Products to Search for Potential New Drugs in the Treatment of Parkinson’s Disease

In idiopathic Parkinson’s disease, the identity of the neurotoxin that triggers the mechanisms involved in the loss of neuromelanin-containing dopaminergic neurons is unknown. It has been proposed that aminochrome could be the neurotoxin that triggers all of these neurotoxic mechanisms mentioned above, and it does not have a propagating character.

**Figure 2:**
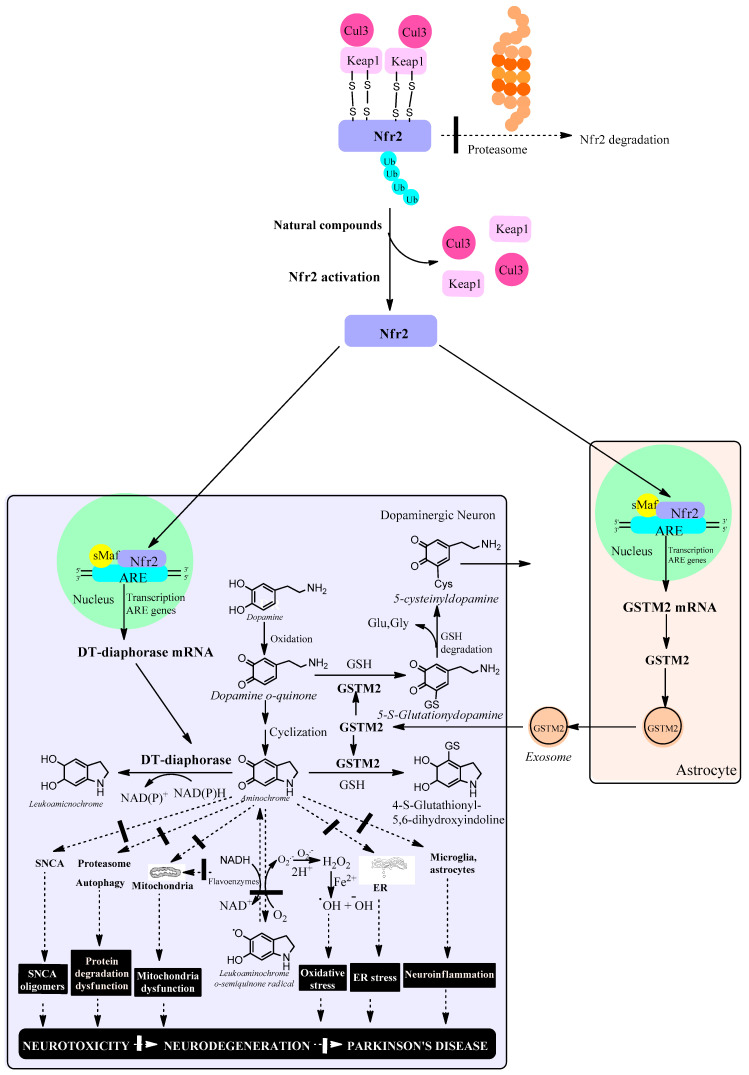
Natural compounds activate the KEAP1/Nrf2 signaling pathway, which increases the expression of antioxidant enzymes such as DT-diaphorase in dopaminergic neurons and glutathione transferase M2-2 (GSTM2) in astrocytes. In neuromelanin-containing dopaminergic neurons, Nfr2 increases the expression of DT-diaphorase, which prevents neurotoxic effects of aminochrome such as the formation of neurotoxic oligomers of alpha-synuclein, dysfunction of protein degradation systems, mitochondrial dysfunction, oxidative stress, endoplasmic reticulum stress and neuroinflammation. In astrocytes, Nfr2 increases the expression of GSTM2, which is excreted through exosomes that penetrate neuromelanin-containing dopaminergic neurons. Within this neuron, GSTM2 is released, conjugating aminochrome to 4-S-glutathionyl 5,6-dihydroxindoline, which is resistant to biological oxidants such as hydrogen peroxide, superoxide and dioxygen. In addition, GSTM2 conjugates dopamine o-quinone to 5-glutathionyldopamine, which is enzymatically degraded to 5-cysteinyldopamine, a metabolite that has been found in human neuromelanin and cerebrospinal fluid, suggesting that this metabolite is a final product. Cullin 3 E3 ubiquitin ligase (Cul3); Kelch-like ECH-associated protein 1 (KEAP1); ubiquitin (ub); E2-related factor 2 (Nfr2); small musculoaponeurotic fibrosarcoma (sMaf); antioxidant responsive element (ARE); endoplasmic reticulum (ER); reduced glutathione (GSH); glutamate (glu); glycine (gly); alpha-synuclein (SNCA); hydrogen peroxide (H_2_O_2_).

The role of phase 2 enzymes is to deactivate and detoxify molecules that have been activated by phase-1 enzymes of drug metabolism with the goal of eliminating them from the body. Within the group of phase 2 enzymes we have antioxidant enzymes that increase their expression by activating the KEAP1/Nrf2 signaling pathway. However, activation of the KEAP1/Nrf2 signaling pathway increases the expression of DT-diaphorase and glutathione transferase M2-2 enzymes which prevents neurotoxic effects of aminochrome such as mitochondrial dysfunction, the formation of neurotoxic alpha-synuclein oligomers, oxidative stress, neuroinflammation, endoplasmic reticulum stress plasma reticulum stress and protein degradation dysfunction (Figure 2).

If we consider that the degenerative process of idiopathic Parkinson’s disease depends on a degenerative model such as single neuron degeneration that is triggered by the neurotoxic action of aminochrome, efforts to find a new drug that stops or slows the progress of the disease should be directed towards finding molecules that increase the expression of the enzymes DT-diaphorase and glutathione transferase M2-2.

The disadvantage of single neuron degeneration is the difficulty of being used in an animal model. Unfortunately, it is impossible to use aminochrome as a preclinical model in an animal since the technology does not exist to inject a single neuron. Therefore, the only possibility to search for new molecules that may have a therapeutic effect in a single-neuron degeneration model is to search for molecules that (i) increase the expression of DT-diaphorase and Glutathione transferase M2-2 through activation of the KEAP1/Nrf2 signaling pathway; and (ii) these molecules prevent the neurotoxic effects of aminochrome in cell cultures.

## 8. Conclusions

The possible use of phytocompounds in the treatment of Parkinson’s disease has been studied in preclinical models. Plant derivatives such as kurarinone, *Ginkgo biloba* L., curcumin, *Hibiscus asper* Hook. f., berberine [113] and *Carthamus tinctorius* L. have been shown to have a positive effect in preclinical models of Parkinson’s disease [114,115,116,117,118,119,120]. The failure of a series of clinical studies of potential molecules intended to stop or slow the progress of the degenerative process in idiopathic Parkinson’s disease is one of the largest problems for patients and research groups. In our opinion, the reason for the failure depends on the use of preclinical models based on exogenous neurotoxins that do not represent what happens in idiopathic Parkinson’s disease because they use massive and very rapid degenerative models that contrast with the extremely slow progress of the disease, which takes years for the appearance of motor symptoms and their subsequent progress. We have proposed a single-neuron model of degeneration in which an endogenous neurotoxin generated within the same neuron induces a non-expansive neurodegenerative process, which only induces the loss of this single neuron without affecting neighboring neurons. To date, aminochrome is the only endogenous neurotoxin we know of that is formed within neuromelanin-containing dopaminergic neurons and does not induce expansive neurodegeneration. The increased expression of DT-diaphorase and glutathione transferase M2-2 enzymes and other antioxidant enzymes is mediated by the activation of the KEAP1/Nrf2 signaling pathway. However, only DT-diaphorase and glutathione transferase M2-2 prevent the neurotoxic effects of aminochrome in neuromelanin-containing dopaminergic neurons. Given the technical impossibility of injecting aminochrome into a single neuron, the only alternative is to search for new molecules such as natural compounds that both increase DT-diaphorase and glutathione transferase M2-2 expression by activating the KEAP1/Nrf2 signaling pathway and prevent the neurotoxic effects of aminochrome in cell cultures.

## Figures and Tables

**Table 1:** Natural compounds that activate KEAP1/Nrf2 signaling pathway. DT-diaphorase (NQO1); Hemeoxigenase-1 (HO-1); γ-glutamylcysteine-synthetase (γGCS); heme oxygenase 1 (HO-1); NAD(P)H quinone oxidoreductase 1 (NQO1); superoxide dismutase-1 (SOD-1); catalase (CAT), superoxide; glutathione peroxidase (GPx), endothelial and inducible nitric oxide synthases (eNOS and iNO), glutathione transferase (GST); glutathione transferase M2-2 (GSTM2).

Compound	Model	Increased Enzymes	Methodology (Nrf2)	Reference (N°)
Hyperoside	Testicular injury Renal cells	CAT, Mn-SOD, HO-1, NQO1 NQO1	Western blot Western blot	[67] [68]
Sulforaphane	Rotenone animal Keratinocytes	HO-1, NQO1, HO-1, NQO1, γGCS	Western blot mRNA level	[69] [70]
Resveratrol	PC12 cells Neural stem cells	HO-1 HO-1, NQO1	Western blot Western blot	[72] [73]
Curcumin	PC12 cells Corneal endothelial cells Kunming mice	HO-1 SOD-1, HO-1 HO-1,NQO1, γGCS	Western blot Nuclear/cytosol fractionation kit Western blot	[74] [75] [76]
Quercetin	Human HepG2 cells	NQO1	Western blot	[77]
Epigallocatechin gallate	Mammary epithelial cells	HO-1, SOD-1	Western blot	[72]
*Withania somnifera*	Primary human umbilical vein endothelial cells	HO-1	Western blot	[79]
Thonningianin A	SH-SY5Y cells	HO-1	Western blot Molecular docking	[80]
Berberine	Male rats	SOD-1	q-RT-PCR	[81]
Byproduct grape seed meal	IPEC-1 cells	CAT, SOD-1, GPx, eNOS, iNO	q-RT-PCR	[84]
Hederagenin	Extracellular matrix	HO-1, NQO1	Western blot	[83]
Isoglycyrrhizinate	KunMing mice	CAT, SOD-1	Western blot	[84]
*Clostridium butyricum*	IPEC-J2) cells	SOD-1, GPx	siRNA	[85]
Formononetin	C57BL/6J mice HK-2 cells	HO-1, NQO1 HO-1, NQO1	Nrf2 knockout Western blot	[86] [87]
Physalin H	RAW264.7 cells	HO-1, NQO1	qRT-PCR, western blot	[88]
Icarin	Diabetes mellitus rats	HO-1	Western blot	[89]
Oxymatrine	Primary cardiac fibroblasts	HO-1	qRT-PCR, siRNA	[90]
*Coptis chinensis*	HepG2 cells	GST, NQO1, HO-1	Western blot	[91]
Withaferin A	Endothelial cells	HO-1	Western blot, siRNA	[79]
Callicarpa kwangtungensis	RAW 264.7 macrophages	HO-1, NQO1	Western blot	[95]
Oxyresveratrol	Hepatocytes	HO-1, NOQ1	Western blot	[96]
Monoammonium glycyrrhizinate	Hepatic injury	HO-1, NOQ1	Western blot	[97]
Shufeng Jiedu	LPS-induced acute lung injury	HO-1, NQO1	Western blot	[98]
Ginnalin A	SH-SY5Y cells	HO-1, NQO1	Western blot, qRT-PCR	[99]
S-allylmercaptocysteine	LPS-induced acute lung injury	HO-1, NQO1	Western blot	[100]
Crocin	Male rats	HO-1, NQO1	Western blot	[103]
Chalcone xanthohumol	PC12 cells	NQO1	Nrf2 knockdown	[101]
Safranal	Rotenone	HO-1,GST, NQO1	Western blot	[108]
Astaxanthin	Increase	HO-1, NQO1, GSTM2	qRT-PCR	[109]
Docosahexaenoic acid	Increase	HO-1, NQO1, GSTM2	qRT-PCR	[109]
Eicosapentaenoic acid	Increase	HO-1, NQO1, GSTM2	qRT-PCR	[109]
Thonningianin A	SH-SY5Y cells	HO-1	Immunofluorescence	[80]
